# Right Bochdalek congenital diaphragmatic hernia: a tertiary center’s experience over 13 years

**DOI:** 10.1186/s43159-021-00081-z

**Published:** 2021-05-05

**Authors:** Alshaima Alghamdi, Enaam Raboe

**Affiliations:** King Fahd Armed Forces Hospital, Jeddah, Saudi Arabia

**Keywords:** Right diaphragmatic hernia, Congenital diaphragmatic hernia, Bochdalek diaphragmatic hernia

## Abstract

**Background:**

Right Bochdalek congenital diaphragmatic hernia (RB-CDH) is far less common than left Bochdalek congenital diaphragmatic hernia, accounting for only 13% of cases. There are limited published data on the outcomes and survival rate of RB-CDH.

We aimed at investigating the clinical characteristics and analyzing the risk factors of survival in neonates with RB-CDH treated in our center over a period of 13 years.

**Results:**

Fifteen infants with RB-CDH were identified. Most of the patients were full term (74%). The mean birth weight was 2.90± 0.72 kg. The ratio of male to female was 2:1. The mean APGAR score at 1 min was 5.31±2.34, and 7.30±1.59 at 5 min. Ten patients (67%) were imaged by antenatal ultrasound. Eleven patients (73.33%) survived to go for surgical repair. The hernia sac was found in 5 patients (45%). Most hernial defects were closed in a primary fashion. The mean age at the operative repair was 8.11±9.90 days. The average NICU stay for all patients was 40.47±50.38 days. The mean follow-up period was 20.45±9.34 months. Three patients had postoperative complications. The total survival rate in neonates with RB-CDH was 9/15 (60%). Nine out of 11 (82%) neonates survived after surgical repair. Four patients (27%) died before surgical repair. Ventilation-related bilateral pneumothorax was a contributing cause of death in three patients. Birth weight was found lower in the non-survivor’s group (*P* < 0.05). Moreover, the degree of pulmonary hypertension was more severe among non-survivors. No statistical significance was observed between other variables and mortality.

**Conclusion:**

We found that low birth weight and the presence of severe PHTN were risk factors for mortality in neonates with RB-CDH. These results are in line with previous studies on prognostic factors in CDH. Ventilator-related pneumothorax appears to be a significant contributing cause of death. Long-term follow-up studies of infants born with RB-CDH are needed as small number of cases limits large-volume RB-CDH studies.

## Background

Congenital diaphragmatic hernia (CDH) represents a rare developmental defect, with an estimated prevalence of 2-4 per 10,000 live births in the USA and Europe [1, 2]. While a growing proportion of cases are diagnosed prenatally, some are diagnosed at birth due to immediate respiratory distress.

The diaphragm develops from the fusion of four embryonic components [3]. The posterolateral (Bochdalek) diaphragmatic hernia accounts for 90% of all diaphragmatic hernia cases. The right Bochdaleck congenital diaphragmatic hernia (RB-CDH) is less common than the left Bochdaleck congenital diaphragmatic hernia (LB-CDH) (13% vs 85%, respectively) [4].

Pulmonary hypertension (PHTN) occurs in up to 75% of infants with CDH [5]. The severity of pulmonary hypoplasia and hypertension are the major determinants of overall survival in those patients [6, 7].

The mortality of CDH neonates remains high despite neonatal intensive care improvements [8]. The reported 24-h mortality ranges between 21–31%, whereas the 1-year mortality can be as high as 46% [9]. There are limited published literature on the outcomes and associations of RB-CDH.

### Aim

Our study aimed at investigating the clinical characteristics and analyzing the risk factors of survival in neonates with RB-CDH treated in our center over a period of 13 years.

## Methods

We conducted a retrospective study of all infants with RB-CDH treated at our tertiary care hospital between January 2005 and January 2018. The inclusion criterion was a diagnosis of RB-CDH. Infants who had diaphragmatic eventration were excluded.

The diagnosis of RB-CDH was made by prenatal ultrasonographic scans, plain chest radiography films, or contrast-enhanced computerized tomography in doubtful cases. Cardiac malformations were diagnosed using two-dimensional (2D) echocardiography.

The following variables were assessed: gestational age, mode of delivery, Apgar score at 1 and 5 min, birth weight, associated anomalies, duration of mechanical ventilation, and the severity of pulmonary hypertension, in addition to the surgical approach, timing of repair, need for a surgical patch, postoperative complications, and recurrence of hernia.

Data are presented as an absolute number with a percentage or mean ± standard deviation. A univariate analysis was performed to describe demographic and perioperative data. A comparison between two groups (group A = survivors/group B = non-survivors) was performed using a Student’s *t* test for continuous variables and Fisher’s exact test for categorical variables. A *P* value <.05 was defined as significant. The statistical analysis was performed using the statistical software (IBM SPSS Statistics for Windows, Version 22.0., IBM Corp, Armonk, NY, USA).

## Results

**Fig. 1:**
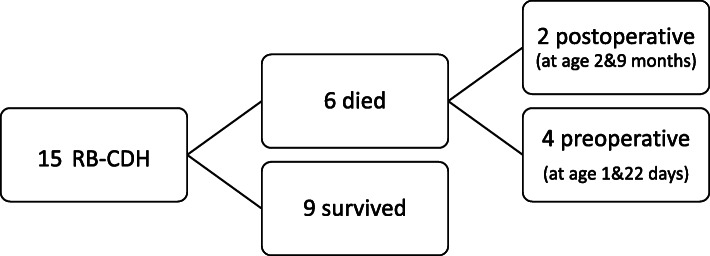
Flow diagram of infants with RB-CDH

We identified 15 infants with RB-CDH during the study period (Fig. 1). Most of the patients were full term (74%). The mean birth weight was 2.90± 0.72 kg. There were 10 males (67%) and 5 females (33%) with a male to female ratio of 2:1. Seven newborns (47%) were delivered by cesarean section, and eight were delivered vaginally (53%). The mean APGAR score was 5.31±2.34, and 7.30±1.59 at 1 min and 5 min, respectively.

All patients were diagnosed from prenatal ultrasound and/or radiological imaging after birth, with the clinical signs of tachypnea, apnea, bradycardia, desaturation to Spo2 less than 90% and scaphoid abdomen. Ten patients (67%) were diagnosed by antenatal ultrasound. RB-CDH was found only in 6 fetuses (40%), 2 fetuses (13%) had signs of hydrops (pleural effusion, pericardial effusion, and ascites), and 2 (13%) had only polyhydramnios. All newborns were diagnosed by chest X-ray, but four of them needed chest CT scans to confirm the diagnosis.

More than quarters (27%) of the parents were consanguineous. Nine mothers did not have any medical illness (60%), whereas four (27%) had DM, VSD, or hypothyroidism. Two mothers (13%) had multiple abortions. Eleven patients survived to undergo repair with laparotomy. One patient needed an additional right thoracotomy for hepatopulmonary fusion. The liver was the most frequently herniated organ—found in 10 patients (91%)—followed by small bowel (82%) and large bowel (36%). The hernial sac was found in five patients (45%).

Most hernial defects were closed primarily using either polypropylene, silk, or polyester sutures. Polytetrafluoroethylene (PTFE) mesh was used in one patient with a large defect and transversus abdominis muscle flap was fashioned to close the defect in another patient. The mean age at the operative repair was 8.11±9.90 days. The average NICU stay for all patients was 40.47±50.38 days.

The mean follow-up period was 20.45±9.34 months. Three patients had postoperative complications, two had an incisional hernia, and one had GERD. One patient had recurrences twice, which mandated mesh repair at the age of 6 months. The total survival rate in neonates with RB-CDH was 9/15 (60%), and two (18%) died postoperatively from severe persistent pulmonary hypertension of the newborn (PPHN) and cardiac complications at the age of 2 months and 9 months, respectively. Four patients (27%) died early after birth (before the surgical repair), three of them died on the first day of life, and one died at the age of 22 days. Ventilation-related bilateral pneumothorax was a contributing cause of death in three patients. Nine out of eleven (82%) neonates survived after surgical repair.

When a comparison was made among the survivors (*n* =9) and non-survivors (*n* =6) (Table 1), birth weight was found lower in non-survivors, which was statistically significant (*P* < 0.05). Moreover, the degree of pulmonary hypertension was more severe among non-survivors. There was no statistically significant difference in gestational age, Apgar score, and cardiac malformations between RB-CDH survivors 9/15 (60%) and non-survivors 6/15 (40%). The details of cardiac abnormalities are shown in Table 2. Though most of the dead patients required HFOV (83.33%), the result was not statistically significant compared to only 44.44% of the survivors. Two of the survivors had associated non-cardiac anomalies (undescended testicle, grade1 hydronephrosis, and hydrocephalus). It was noticed that the average number of days of mechanical ventilation (HFOV and conventional ventilation) in the survivor group was 14.33±9.77days.

**Table 1:** Comparison between survivors and non survivors of right-sided congenital diaphragmatic hernia patients

	Survivors (9/15)	Non-survivors (6/15)	*P* value
**Gestational age (weeks)**	-Mean 38.56±2.19 7 full term (77.78%) 2 preterm (22.22%)	-Mean 35.67±4.03 3 full term (50%) 3 preterm (50%)	.093
**Birth weight (kg)**	-Mean 3.32±0.45 kg	-Mean 2.35±0.78 kg	.008
**Apgar score (out of 10)**	-Mean at 1 min: 5.67±2.79	-Mean at 1 min: 5±1.55	.604
	-Mean at 5 min: 7.33±1.50	-Mean at 5 min: 7±1.55	.683
**Cardiac malformations**	-5 newborns (55.56%): 4 PDA 3 ASD/PFO 1 VSD 1 pulmonary artery stenosis	-5 newborns (83.33%): 4 ASD/PFO 3 PDA 3 right side aortic arch 1 VSD 1 pulmonary artery stenosis 1 double outlet right ventricle	.580
**Pulmonary hypertension (estimated from echocardiography done at first week of life)**	-6 newborns (66%) had mild PHTN. -3 newborns (33%) had moderate to severe PHTN.	-All (100%) had signs of severe PHTN^a^.	.027
**Need for HFOV**^**b**^ **and duration**	4 needed HFOV (44.44%) (mean 8±3.5, range 5-14 days)	5 needed HFOV (83.33%) (mean 2.2±2.4, range 1-7 days)	.286

^a^Severe pulmonary hypertension (PHTN): estimated pulmonary arterial pressure that is equal to systemic blood pressure or more (suprasystemic)

^b^*HFOV* high frequency oscillatory ventilation

**Table 2:** Spectrum of congenital heart disease (CHD), degree of pulmonary hypertension (PHTN), and patient outcome

Infant details	Type of CHD and degree of PHTN	Outcome
Infant 1	-Left pulmonary artery stenosis, PDA -Mild pulmonary hypertension	Survived
Infant 2	-Small muscular VSD, moderately dilated right ventricle -Moderate to severe pulmonary hypertension	Survived
Infant 3	-Small ASD, moderate PDA -Mild pulmonary hypertension	Survived
Infant 4	-Mild tricuspid regurgitation, large PDA, PFO, mild dilated right atrium -Moderate to severe pulmonary hypertension	Survived
Infant 5	PDA, small PFO, mild tricuspid regurgitation -Moderate to severe pulmonary hypertension	Survived
Infant 6	-Severe pulmonary artery stenosis, PDA, PFO, VSD, double outlet right ventricle, right aortic arch -Severe pulmonary hypertension	Died (at age of 9 months)
Infant 7	-ASD -Severe pulmonary hypertension	Died (at age of 1 day)
Infant 8	-ASD -Severe pulmonary hypertension	Died (at age of 1 day)
Infant 9	-ASD, dilated right ventricle and atrium, large PDA -Severe pulmonary hypertension	Died (at age of 1 day)
Infant 10	-Right sided aortic arch, moderate PDA, moderate tricuspid regurgitation -Severe pulmonary hypertension	Died (preoperatively at age of 22 days)

*PDA* patent ductus arteriosus, *ASD* atrial septal defect, *PFO* patent foramen ovale, *VSD* ventricular septal defect

## Discussion

CDH has been recognized as a syndrome, including diaphragmatic defects, pulmonary hypoplasia, and pulmonary hypertension [10, 11]. Some authors have observed a female predominance for a Bochdalek hernia; however, others have observed a male predominance [12]. In our study, also male preponderance was noted in RB-CDH.

Neonates with CDH are at risk for prematurity and low birth weight (LBW), which predisposes them to increased mortality [13, 14]. Grover et al. reported that the mortality rate of preterm neonates with CDH (<34 weeks or birth weight <2000 g) was 50%, compared to 27% mortality rate for neonates >34 weeks [15]. In this study, 5 of our patients (33%) were preterm (<37weeks), 3 of them (60%) died. We found no significant difference in mean gestational age at birth between survivors and non-survivors.

CDH is usually diagnosed immediately after birth with signs of respiratory distress and mediastinal shift on chest plain film [16]. All patients had respiratory distress features at birth, which is related to uncorrectable pulmonary hypoplasia and potentially reversible pulmonary hypertension [16].

A low Apgar score is usually associated with severe asphyxia and is a major independent predictor of mortality rate. However, our results suggest that the non-survivor group did not have statistically significant lower Apgar scores compared to the survivor group.

Early prenatal diagnosis is important, as the mother should be referred to a tertiary care center for optimal care before birth. Most CDH defects are detected after 16 weeks of gestation. The prenatal detection rate varies in published studies, from 50 to 70% [17]. However, prenatal detection of CDH is uncommon in developing countries due to inadequate facilities and poor patient compliance [12]. In our study, 6/15 (40%) of the cases were diagnosed prenatally as RB-CDH. Three-dimensional estimation of the total fetal lung volume (TFLV), calculation of lung to head ratio (LHR), observed to expected lung head ratio (O/E LHR), and calculation of the lung to thoracic circumference ratio have been widely used as prognostic indicators [18, 19]. This study’s limitation is the unavailability of the antenatal ultrasonographic measurements (LHR and TFLV) in the medical records.

Ramakrishnan and colleagues found that isolated CDH cases had better neonatal and 1-year survival rates, and chromosomal cases were associated with the worst survival rates [20]. Cardiac malformations are the most common associated anomalies. In a review of 4268 infants with CDH, approximately 18% of infants had an associated cardiac defect. Major cardiac lesions (excluding PFO, ASD, PDA) were associated with overall survival of 36% compared to infants with minor anomalies (67% survival) and those without cardiac defects (73% survival) [21]. In our review, none of the neonates had associated syndromes. Among the nonlethal cardiac malformations, ASD, VSD, and PFO predominated, similar to the findings reported by Sweed Y and colleagues [22].

Identification and management of pulmonary hypertension are critically important in the newborn period. CDH-associated pulmonary hypertension was graded—by echocardiography—into mild, moderate, and severe based on pressure gradient through ductus arteriosus. In our study, all non-survivors had signs of severe PHTN, whereas 3 out of 9 survivors (33.33%) had moderate to severe PHTN, with a *P* value of .027, which was statistically significant.

The concept of gentle ventilation strategies (permissive hypercapnia) was introduced by Wung and colleagues in their 1995 retrospective, nonrandomized study to reduce iatrogenic lung injury from barotrauma; this ventilation strategy has been employed in most centers, including our center [23]. HFOV has also been utilized in the perinatal management of CDH to reduce pulmonary barotraumas. In this study, all patients received mechanical ventilation. Four out of nine (44.44%) survivors and 5/6 (83.33%) non-survivors received HFOV, with a *P* value of .286, which was statistically insignificant. HFOV was used for patients with refractory hypercapnia or high peak inspiratory pressures, and only one neonate required it postoperatively. HFOV may be a more effective mode of ventilatory support than conventional ventilation when used as an initial mode of therapy [24]. Extracorporeal membrane oxygenation (ECMO) is used if HFOV fails to maintain the goal physiologic parameters. Inhaled nitric oxide (iNO) was initiated at 20 ppm for all patients receiving HFOV. Usui et al. reported 14% incidence of pneumothorax among 510 neonates with CDH [25]. In our study, none of the neonates who had pneumothorax survived.

Delayed surgical repair is the current management in most of the surgical centers. Preoperative stabilization aims at optimizing the respiratory function and allowing full clinical and cardiac assessment. We operated on 11 RB-CDH cases via an upper transverse or subcostal abdominal incision. We used polypropylene mesh in one patient which had a big defect size. Unfortunately, he did not survive. Minimally invasive approach has the advantage of decreased postoperative pain; however, the recurrence rate appears to be higher than with open techniques, and the patient should have stringent intraoperative monitoring of ETCO2 and PaCO2 [15].

The presence of hernia sac with CDH—which was reported in 20% of patients—can improve the prognosis in affected neonates [26]. Intraoperatively, we found a hernia sac in 5/11 (45.5%) neonates; all of them survived.

Liver herniation into the chest is considered a poor prognostic sign. Fibrous fusion between the liver and the lung has been reported [27]. We observed hepatopulmonary fusion in one patient which required thoracotomy in addition to right subcostal incision. These anatomic findings can significantly complicate the diaphragmatic defect repair.

Recurrent diaphragmatic hernia is a dominant surgical challenge. The reported recurrence rate is 15% in the first couple of years following repair. One CDHSG registry review found that large hernial defect and the use of a minimally invasive surgery were associated with early recurrence after CDH repair [28]. Recurrent hernias may occur in up to 28% of infants undergoing mesh repairs and in 2% of primary repairs [29]. In our study, we encountered one patient with recurrent hernia who presented with vomiting at 40 days old and 9 months of age, which was successfully repaired and is doing well in 3 years follow-up.

The overall survival rate of CDH neonates in NICU ranges from 21 to 83% according to various recent studies [30]. Multiple studies from developing countries reported 50%–65% rate of survival for CDH patients [31]. Recent studies from developed countries have shown improved survival rate for isolated CDH up to 85%–90% [32].

Several studies have shown that RB-CDH is associated with higher mortality than LB-CDH [33, 34]. Burgos and colleagues explained that by the higher proportion of larger defects in this group compared to LB-CDH. Therefore, it seems that the hernial defect size is important for the outcome of CDH [35]. However, other reports did not find a statistical difference in mortality rate when compared to left-sided defects [36, 37]. A recently published French national cohort study found that patients with RB-CDH did not have a higher risk of mortality than LB-CDH [38]. Another retrospective multicenter study compared characteristics and outcomes of neonates with RB-CDH vs. LB-CDH and found no difference in mortality or hernia recurrence [39].

In a study by Aihole and colleagues, a quarter of neonates (25%) with RB-CDH died preoperatively [40]. In our study, 4/15 (27%) of RB-CDH neonates did not survive to the time of surgical repair, and 2 died post repair while 9/11 (82%) of neonates survived postoperatively with total mortality of 40% (4 preop and 2 postop). Interestingly, Thomas et al. found infants with RB-CDH had a better survival than those with LB-CDH [41].

## Conclusion

We found that low birth weight and the presence of severe PHTN were risk factors for mortality in neonates with RB-CDH. These results are in line with previous studies on prognostic factors in CDH. Ventilator-related pneumothorax appears to be a significant contributing cause of death. Long-term follow-up studies of infants born with RB-CDH are needed as large-volume RB-CDH studies are limited by small numbers of cases.

## Data Availability

The datasets used and/or analyzed during the current study are available from the corresponding author on reasonable request.

## Abbreviations

CDH: Congenital diaphragmatic hernia
RB-CDH: Right Bochdaleck congenital diaphragmatic hernia
LB-CDH: Left Bochdaleck congenital diaphragmatic hernia
PHTN: Pulmonary hypertension
CT scans: Computerized tomography scans
DM: Diabetes mellitus
VSD: Ventricular septal defect
PTFE: Polytetrafluoroethylene
GERD: Gastroesophageal reflux disease
PPHN: Persistent pulmonary hypertension of the newborn
HFOV: High frequency oscillatory ventilation
ECMO: Extracorporeal membrane oxygenation
TFLV: Total fetal lung volume
LHR: Lung to head ratio
O/E LHR: Observed to expected lung head ratio
PFO: Patent foramen ovale
ASD: Atrial septal defect
PDA: Patent ductus arteriosus
iNO: Inhaled nitric oxide
ETCO2: End-tidal CO2
PaCO2: Partial pressure of carbon dioxide
CDHSG: Congenital diaphragmatic hernia study group
NICU: Neonatal intensive care unit
